# Influence of Climatic Factors on Human Hantavirus Infections in Latin America and the Caribbean: A Systematic Review

**DOI:** 10.3390/pathogens11010015

**Published:** 2021-12-23

**Authors:** Kirk Osmond Douglas, Karl Payne, Gilberto Sabino-Santos, John Agard

**Affiliations:** 1 Centre for Biosecurity Studies, Cave Hill Campus, The University of the West Indies, Cave Hill, St. Michael BB11000, Barbados; 2 Centre for Resource Management and Environmental Studies, Cave Hill Campus, The University of the West Indies, Cave Hill, St. Michael BB11000, Barbados; karl.payne@cavehill.uwi.edu; 3 School of Public Health and Tropical Medicine, Tulane University, 1324 Tulane Ave Suite 517, New Orleans, LA 70112, USA; gsabino@tulane.edu; 4 Centre for Virology Research, Ribeirao Preto Medical School, University of Sao Paulo, 3900 Av. Bandeirantes, Ribeirao Preto 14049-900, SP, Brazil; 5 Department of Life Sciences, The University of the West Indies, St. Augustine 999183, Trinidad and Tobago; John.Agard@sta.uwi.edu

**Keywords:** climate change, hantavirus, Latin America, Caribbean, public health, biosecurity, rainfall, temperature, infectious diseases, vector-borne

## Abstract

Background: With the current climate change crisis and its influence on infectious disease transmission there is an increased desire to understand its impact on infectious diseases globally. Hantaviruses are found worldwide, causing infectious diseases such as haemorrhagic fever with renal syndrome (HFRS) and hantavirus cardiopulmonary syndrome (HCPS)/hantavirus pulmonary syndrome (HPS) in tropical regions such as Latin America and the Caribbean (LAC). These regions are inherently vulnerable to climate change impacts, infectious disease outbreaks and natural disasters. Hantaviruses are zoonotic viruses present in multiple rodent hosts resident in Neotropical ecosystems within LAC and are involved in hantavirus transmission. Methods: We conducted a systematic review to assess the association of climatic factors with human hantavirus infections in the LAC region. Literature searches were conducted on MEDLINE and Web of Science databases for published studies according to Preferred Reporting Items for Systematic reviews and Meta-Analyses (PRISMA) criteria. The inclusion criteria included at least eight human hantavirus cases, at least one climatic factor and study from > 1 LAC geographical location. Results: In total, 383 papers were identified within the search criteria, but 13 studies met the inclusion criteria ranging from Brazil, Chile, Argentina, Bolivia and Panama in Latin America and a single study from Barbados in the Caribbean. Multiple mathematical models were utilized in the selected studies with varying power to generate robust risk and case estimates of human hantavirus infections linked to climatic factors. Strong evidence of hantavirus disease association with precipitation and habitat type factors were observed, but mixed evidence was observed for temperature and humidity. Conclusions: The interaction of climate and hantavirus diseases in LAC is likely complex due to the unknown identity of all vertebrate host reservoirs, circulation of multiple hantavirus strains, agricultural practices, climatic changes and challenged public health systems. There is an increasing need for more detailed systematic research on the influence of climate and other co-related social, abiotic, and biotic factors on infectious diseases in LAC to understand the complexity of vector-borne disease transmission in the Neotropics.

## 1. Introduction

Hantaviruses are single-stranded (SS) negative-sense RNA viruses from the *Hantaviridae* family, order *Bunyavirales* [1]. The typical virions range from 80–120 nm in diameter but atypical elongated virions 170 nm in length can occur [2,3]. The hantavirus genome is comprised of three RNA segments, the Small (S) segment which encodes the nucleocapsid protein (N), the Medium (M) which encodes the precursor polyprotein that yields glycoproteins Gn (formerly G1), Gc (formerly G2) and the Large (L) segment which encodes the L protein or viral transcriptase/replicase [4,5]. Members of the family *Hantaviridae* are classified into seven genera accordingly with their particular evolutionary history and distinct host-reservoir: *Actinovirus* (fish), *Aganathorvirus* (jawless fish), *Loanvirus* (bat), *Mobatvirus* (bat or mole), *Orthohantavirus* (rodent), *Thottimivirus* (shrew) and *Reptillovirus* (reptile) [1]. Thus far, members of the genus *Orthohantavirus* are the only known hantavirus species to cause clinical disease in humans through aerosolized virus particles present in rodent excreta, by direct contact with rodent blood or bites [1,6]. 

Hantavirus infections are an emerging infectious disease and a zoonotic threat in the Latin America and the Caribbean (LAC) region. Hantavirus infection causes hantavirus pulmonary syndrome (HPS) or hantavirus cardiopulmonary syndrome (HCPS) and haemorrhagic fever with renal syndrome (HFRS). It is estimated that 150,000 to 200,000 annual hantavirus cases occur globally [7]. The endemicity of other infectious diseases with similar clinical symptomology to hantavirus disease can make accurate clinical diagnosis of febrile acutely infected patients in the LAC region difficult. Dengue virus, Chikungunya virus, Mayaro virus, Zika virus and *Leptospira* infections are all endemic to both Latin America and the Caribbean. These regions consist of rich biodiversity hotspots (especially Neotropical rodents) and Neotropical climates that can result in new knowledge of hantavirus transmission that is different from the traditional and better-known hantavirus transmission models in Europe, Asia and North America [8]. The links to cyclic rodent populations and hantavirus disease outbreaks have been established in Europe with bank voles. 

The first evidence of hantavirus infections in the Caribbean was observed in Barbados among human cases suspected of leptospirosis and also in rodents (*Rattus* spp.) but the identity of the hantavirus strain(s) remain(s) unknown [9]. Later studies provided further evidence of hantavirus circulation in other countries within the region including Grenada, Trinidad & Tobago, and a single exported human case from Cuba [10,11,12,13]. In Latin America, instances of human-to-human transmission with a hantavirus strain, the Andes hantavirus (ANDV), were observed among HCPS cases in Argentina and Chile [14,15,16]. To date, there are two main species of hantavirus in South America: *Andes orthohantavirus* (ANDV), considered one of the most lethal hantaviruses, and *Laguna Negra orthohantavirus* (LANV) [1]. However, these species possess great diversity and represent the highest diversity observed in the world [17,18]. Within ANDV, five genotypes have been described: *Araraqurara orthohantavirus* (ARQV), *Castelo dos Sonhos orthohantanvirus* (CASV), *Juquitiba orthohantavirus* (JUQV), *Lechiguanas orthohantavirus* (LECV), and *Orán orthohantavirus* (ORNV); and within LANV there are four genotypes: *Anajatuba orthohantavirus* (ANJV), *Maripa orthohantavirus* (MARV), *Rio Mearim orthohantavirus* (RIOMM) and *Rio Mamoré orthothantavirus* (RIOMV) [1,17]. This diversity is due mainly to the distribution of opportunistic rodent-host species that live sympatric, where spillover phenomena favour maintenance and transmission [17]. In LAC regions, rodent hosts of hantaviruses belong to the families Cricetidae and Muridae; however, pathogenic hantaviruses belong mainly to the family Cricetidae, subfamily Sigmodontinae and *Rattus* spp. [6,19,20]. Sigmodontinae rodents are distributed from *Tierra del Fuego*, in South America, to the United States, in North America [19]. These rodents inhabit several types of ecosystems in the Americas, including desert-like biomes, equatorial and tropical forests, swamps, savannas, high altitude fields, and salt marshes [19,20]. Some Sigmodontinae rodents are highly sensitive to ecosystem disruption, while others adapt easily. These rodents are known as opportunistic/generalist and are mainly from the genera *Akodon*, *Calomys*, *Necromys*, *Oligoryzomys* and *Zygodontomys*. Generalist rodents inhabit mostly open vegetated environments utilizing an omnivorous feeding habit which likely influences their successful distribution in disturbed landscapes, especially those converted into agricultural monocultures [21]. Rodent species diversity, distribution and abundance seem to be negatively influenced by the percentage of native forest cover [17,22]. Climate change and environmental degradation increase the contact areas between rodent-hosts and humans, creating a suitable scenario for hantavirus infection [17,18,23]. Other potential hantavirus hosts such as bats, opossums and shrews are present in LAC, thus more research in understanding their potential roles in clinical disease within LAC is needed [23,24,25].

Rainfall patterns in the Caribbean are usually characterized by a dry season (December-May) and a wet season (June-November). The El Niño Southern Oscillation (ENSO), which is associated with a sea surface temperature (SST) gradient anomaly between the eastern Tropical Pacific Ocean and Caribbean Sea, directly affects this rainfall distribution [26,27,28]. El Niño is associated with warmer SST in the central and eastern tropical Pacific Ocean, which has been linked to notable drought events in the Caribbean in 1997–1998, 2009–2010, and 2014–2016 [29,30,31,32,33,34]. Climate change predictions for the LAC region are for more intense hydrometeorological events, which will require adequate preparation by public health authorities for potential hantavirus disease outbreaks, increased transmission and spread.

Climate change can affect the hantavirus mode of transmission and circulation in nature, through impacts on the hantavirus reservoir host rodent population densities [35]. Rodent population dynamics are usually affected by weather and climate. The best example within the Americas is the US Four Corners outbreak in 1993, which was preceded by a dramatic increase in rainfall associated with the 1992–1993 El Niño. This led to increased rodent food resources and a 20-fold increase in the rodent population, followed by the invasion of buildings by rodents, and an increased risk of human hantavirus infections [36,37,38]. The possible effects of climate change may vary from no impact at all to dramatic changes in the size and frequency of hantavirus outbreaks, changes in the spectrum of hantavirus host species, and geographical distribution. These could lead to a major global public health threat. For example, if the consequence of climate change (e.g., increased temperatures) in South America favours the long-tailed pygmy rice rat (*Oligoryzomys longicaudatus*), this could lead to a significant increase in the frequency of human infections by ANDV, found in the mountains of Chile and Argentina, with potentially high mortality rates. ANDV has a high mortality rate (up to 40%) [7,39], and is the only hantavirus strain for which person-to-person transmission has been documented [14,40,41]. Humans become infected primarily through inhalation of contaminated aerosols containing excreta (urine and or feces) from infected rodents [42,43].

To understand the association of various climatic factors with human hantavirus infections, HPS/HCPS and HFRS, we conducted a systematic review to analyse the bibliographic evidence from studies conducted in LAC. This specific type of systematic review has not been previously conducted for LAC. Given the climate predictions for LAC, a greater understanding of the potential impacts of climate change on the dynamics of human hantavirus infections in these regions is strongly desired.

## 2. Results

### 2.1. Bibliographic Search

**Figure 1:**
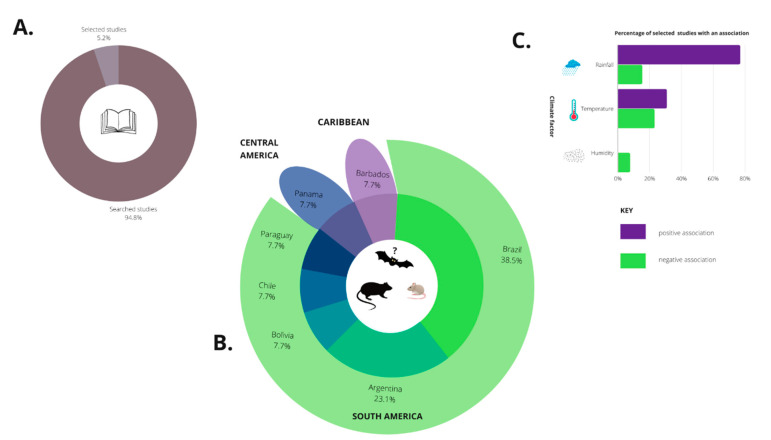
(**A**) The percentages of peer-reviewed studies examining the association of climate factors with human hantavirus infections following bibliographic searches from MEDLINE and Web of Science databases. (**B**) The percentage composition of selected studies of the association of climate factors with human hantavirus infections in the LAC region by country. (**C**) The percentages of selected studies with positive and negative associations of specific climate factors with human hantavirus infections in the LAC region.

To facilitate this systematic review, a detailed bibliographic search was conducted following Preferred Reporting Items for Systematic Reviews and Meta-Analyses (PRISMA) checklist (Box A1) guidelines among the relevant MEDLINE (via PubMed) and Web of Science databases. This resulted in the collection of 236 findings primarily retrieved via MEDLINE via PubMed and 147 findings using the Web of Science database [40]. After application of the relevant inclusion criteria, which included studies involving (1) human hantavirus infections only, available as full-text articles, with relevant abstracts or study titles (with climate and human hantavirus infections), and (2) analysis of exposure to climate factors including temperature, rainfall, humidity, and seasonality, only 13 studies were selected from various LAC countries (Figure 1A and Table 1).

### 2.2. Selected Studies

Studies from seven (7) different LAC countries were included for this systematic review investigating the association of climatic factors with human hantavirus infections (Figure 1B and Table 1). Each selected study was analysed for the associated climatic factor(s) and human hantavirus infections in LAC. Rainfall exhibited the highest positive association with human hantavirus infections (10/13, 76.9%), followed by temperature (4/13, 30.8%). However, temperature exhibited the highest negative association (3/13, 23.1%) with human hantavirus infections, then rainfall (2/13, 15.4%) and finally humidity (1/13, 7.7%). A positive association indicated that human hantavirus cases increased as the climatic factor measure increased. A negative association indicated that there was an inverse relationship between human hantavirus cases and the climatic factor. Other associated co-factors included demographic, socioeconomic, geospatial, and biotic factors. Only one study did not specify the sample size of the human population [41], and five studies did not detail the specific statistical test(s) employed to analyze the results [44,45,46,47,48].

The quality level of selected studies published between 2008 and 2021 was assessed by two independent researchers. An overview of the selected studies is given in Table 1 and the analysis of the metrics used in each study summarised in Table 2. Seven (7) studies obtained a very high quality score (++) [49,50,51,52,53,54], three (3) obtained a high quality score (+) [20,49,55], and three (3) studies obtained a low quality score (−) (Table 1 and Table 2) [46,47,48]. The majority of studies took place in South America–five had been conducted in Brazil [41,44,45,49,54], three in Argentina [50,51,52], and one each in Chile [53], Paraguay [48], and Bolivia [47]. One study was conducted in Central America (Panama) [46] and one in the Caribbean (Barbados) [56]. All selected studies were conducted with various model designs using aggregated data sets. Two studies performed descriptive and Bayesian regressional data analyses [41,45], one study utilised a zero inflated model tailored to count data with excessive zero counts [49], and another used an ecological niche model (ENM) examining the relationships between humans, rodent species and the environments where they occur [44]. Other studies used GLMs and a MaxEnt model [50,51], ARIMA and dynamic regression models [52,53], a cross-sectional epidemiology method [56], and outbreak investigation designs [46,47,48], respectively (Table 1 and Table 2). 

**Figure 2:**
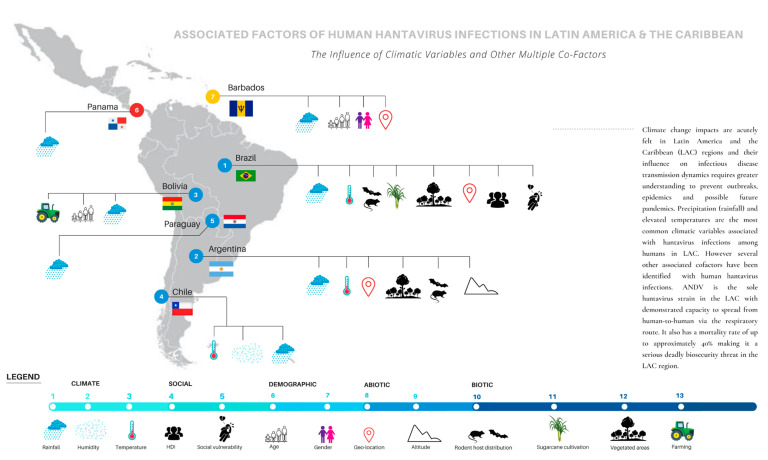
The climatic factors and other co-factors associated with human hantavirus infections in Latin America and the Caribbean. The numbers on the map refer to the specific country where the study or studies was/were conducted.

The time span of study data ranged from one to 23 years, based on monthly, seasonal, or annual climate data with varying time lags (Table 1). Rainfall or precipitation (including snow) was the climatic factor or exposure assessed in all selected studies, while temperature appeared in all selected studies except seven, namely Donaliso [44], Donaliso [45], Andreo [50], Montgomery [47], Bayard [46], Williams [48] and Douglas et al. [56]. Nsoesie et al. [53] analysed relative air humidity. Donaliso et al. [45] investigated winter precipitation as a climatic factor, whilst Prist et al. [54] analysed the Representative Concentration Pathway 4.5 (RCP4.5) and Representative Concentration Pathway 8.5 (RCP8.5) climate scenarios and their associations with human hantavirus infection risk. Wind speed, sunshine, air pressure and other different climate factors were not investigated in any of the selected studies. For assessing the incidence of HPS, HCPS or hantavirus infections, the selected studies relied on national register data (monthly or annual number of reported and confirmed hantavirus cases). Other related co-factors assessed included altitude, HDI, age, gender, rodent host distribution, topography, vegetation, geographical information systems (GIS) location, farming and sugarcane cultivation (Figure 2).

#### 2.2.1. Brazil

There were multiple studies from Brazil (5/13, 38.4%) examining climatic and other related co-factors and human hantavirus infections (Table 1 and Figure 2) [41,44,45,49,54]. Donalisio et al. investigated the influence of spatial distribution and climatic patterns on reported hantavirus cases in São Paulo State, Brazil (n = 80), from 1993 to 2005 [44]. An increase in hantavirus cases during this period may have been due to increased diagnostic capacity and the higher sensitivity of attending physicians. A marked seasonal variation in hantavirus infections was observed in the cerrado (savannah) areas, where a higher incidence occurred in drier months compared to mean rainfall levels in wetter months [44]. The process of harvesting and storing grains increased human exposure to rodents and exposure to aerosols contaminated by rodent excreta. The authors concluded that local transmission risk markers should include both climatic and ecological factors to aid in epidemiological monitoring and disease control.

Another study by Donalisio et al. assessed specific environmental factors and identified areas of hantavirus transmission risk in forested areas in Southern Brazil [45]. The use of an ENM was employed to understand the influence of specific environmental factors including monthly precipitation, monthly minimum and maximum temperatures and bioclimatic factors, land cover and vegetation phenology on 264 HCPS cases between 1993–2008. The notable study limitation was the absence of rodent studies. However, the ENM used offers the opportunity to explore the influence of climatic and landscape factors on HPS over broad regions. This study was useful in illustrating broad areas at elevated risk of hantavirus infections using geospatial factors, rainfall, and vegetative cover. 

Prist et al. examined the associations between annual HCPS incidence and climatic factors (annual precipitation, annual mean temperature), landscape features (native habitat cover, number of forest fragments, area planted with sugarcane), and social factors (number of men older than 14 years and Human Development Index-HDI) across the state of São Paulo, Brazil (1993–2012) [54].

A follow-up study investigated the influence of land use and climatic factors on HCPS incidence in São Paulo, Brazil using HCPS cases from 2000–2010 [41]. The HCPS data was collected from every hospital in the state Sao Paulo [41]. The cases were transformed into binary factors due to the high frequency of zero case counts. A socioeconomic factor, HDI, was added as a cofactor, as was landscape composition or habitat cover including sugarcane cultivation (percentage cover and density of fragments. Future scenarios were predicted for sugarcane expansion and climate change. A Bayesian model was chosen to calculate the probability of HPS case risk in Brazil assessing landscape, social and climatic factors. The landscape was segregated into two biomes: Atlantic and Cerrado given the occurrence of reservoir hosts species and hantavirus strains with each being modelled separately. Model HCPS risk predictions were made using a Bernoulli distribution with seven predictor factors as covariates, namely proportion of sugarcane, proportion of native vegetative patches, human development index (HDI), mean annual temperature, total annual precipitation, and rural male population > 14 years old. Statistical analysis included centering all estimated parameters on their mean and dividing by two standard deviations (SD) for standardization. 

Spatial autocorrelation was assessed using Moran’s I and Queen’s case neighbourhood relation. A non-spatial model was chosen since no spatial autocorrelation was observed. Comparison of the estimated probability of HCPS infections from baseline were modelled with predicted probability in five scenarios: two climate change scenarios, sugarcane expansion and a combination of one climate change scenario and sugarcane expansion. One assumption that used suggested precipitation was not relevant for HCPS risks, however this may not be translatable throughout South America. HCPS risk was categorized as small (<5%), medium (≥5% to ≤10%), high (10 to 20%) and extremely high (>20%) and the assignment of actions was attributed to risk > 5% or medium to extremely high. Current and future HCPS infection risks were predicted and estimations of the number of persons at risk in each scenario were made. 

Temperature was identified as the climatic factor associated with increased HCPS infection risk, whereas climate change scenarios led to increased HCPS infection risk in a specific geospatial region (Midwest region). Considering a sugarcane expansion scenario, the number of persons at risk for HCPS infection increased by 20% for two climate change scenarios RCP4.5 and RCP8.5. 

In conclusion, this study suggested that climate change effects will bear more severe consequences on both HCPS infection risks and the number of persons exposed to hantavirus infection. Climate affects host population densities, food availability, virus survival and ultimately human disease transmission. The influence of precipitation was not considered based on one referenced study due to the absence of a positive association with hantavirus infection risk. However, in several other studies in South America, the converse is true. The strong increase in HCPS infection risk with climate change scenarios also warrants the consideration of the role of precipitation on HCPS infection risk for Brazil. Preventative measures including educational programs, land use management, forest restoration and targeting public health and health care in high-risk areas are notable recommendations to mitigate against the impact of climate change on HCPS infection risk in Brazil.

Muylaert et al. examined the contribution of rodent host diversity, climatic factors, social vulnerability, and land use change on hantavirus disease risk in Brazil [49]. Human notified cases between 1993–2016 from the Ministry of Health were modelled using ENM with several biotic and abiotic factors. Zero inflated modelling was used with geographical precision at the municipal level. Statistical analysis included the use of spatial correlation Moran’s I, temporal term (rw2) + Besag Intrinsic Conditional Auto-Regressive (ICAR) spatial model. Land use change and increased HCPS cases were observed during the study period with a higher concentration of HCPS cases in the Central and South Brazil. In conclusion, increased fauna amounts (maize, forest, and sugarcane) were associated with higher hantavirus disease probability and number of cases per municipality. Temperature, however, showed a negative association with hantavirus cases. Additionally, for the number of HCPS cases, the factors of rainfall, forests and the number of rural workers all showed a positive association.

#### 2.2.2. Argentina

There were three studies (3/13, 23.1%) obtained from Argentina which examined the association of climatic factors on human hantavirus infections (Table 1 and Table 2 and Figure 2).

Andreo et al. (conducted an analysis of HPS and rodent species occurrence (*O. longicaudatus*) in southern Argentina to elucidate the systematic distribution of the risk of HPS transmission [50]. Utilising a species distribution model (SDM) and geographical information system (GIS) software, they estimated areas with the highest human HPS infection risks, specifically regarding the competent rodent host distribution maps. This approach considers the environment (and human factors in describing human infection/exposure risks. This bears relevance as vector-borne diseases often display patterns consistent with the following spatial factors: (1) biotic distribution of flora, fauna (humans and rodent hosts) and microbiota, and (2) abiotic factors, which influence biotic distribution. Previous diagnostic testing of the 149 HPS cases in the study ranging from 1995–2009 indicated that the causative hantavirus was ANDV (the only hantavirus known to cause human-to-human transmission), all of which occurred within four areas of Argentina. The majority of confirmed HPS cases were in Patagonia along the Andes range. Using a GLM, the precipitation-related factors showed a positive association with HPS confirmed cases, whereas temperature related factors showed a negative association [50]. The methods in the study utilized confirmed HPS case data (use of antibody tests), which is one of the study’s main strengths. WorldClim datasets were utilized to produce climatic data layers in this study, but the use of actual climatic data from Argentina would have been more desirable. Two modeling approaches were utilized: GLM with binomial error and a MaxEnt algorithm. This was inclusive of the use of Kruskal-Wallis tests to compare environmental factors with and without HPS, with the significance of factors determined using a *t*-test. Collinearity was evaluated among independent variables using variance inflation factors (VIFs) and pairwise Pearson correlation coefficients. Four intervals of risks based on probability of hantavirus infection were identified. The occurrence of ANDV in Argentina, the only hantavirus strain with the potential of human-to-human spread, makes this study of particular importance.

Ferro et al. examined 902 reported and confirmed HPS cases between 1997–2017 in Northwestern Argentina and their relationship with mean temperature and precipitation using biannual, quarterly, and bimestrial time periods [52]. Lagged temperatures and rainfall (two to six months delay) were significantly associated with HPS cases. This was consistent with the trophic cascade hypothesis indicating that HPS cases followed high precipitation and temperature events. These caused increased food availability for rodent hosts, increased rodent host populations and an increased likelihood of human-rodent contact resulting in higher numbers of human hantavirus infections. The HPS cases included in the study were confirmed by laboratory testing (antibody and molecular tests) with cases that were not reported through the main public health system nor validated by the National Reference Laboratory being excluded, which increased the rigour of this study. Statistical analysis involved the standardization of HPS cases through logarithmic transformation and the use of the Augmented Dickey-Fuller Test. Using dynamic regression, the relationship of HPS prevalence with rainfall and temperature was examined with the ARIMA model and AIC being used to select the appropriate model. The models generated indicated HPS cases were positively and significantly related to rainfall for all time lags but for temperature this was only true for one delay period only. The association of temperature with HPS cases was contradictory, as association was observed with 1 delayed period but inversely associated for 2 delayed periods. This strong association with lagged rainfall and temperature could infer the link of these climatic factors on biotic factors such as food availability for rodent hosts and rodent host population growth.

Vadell et al. examined the relationship between HPS cases and climatic, vegetation, landscape, reservoir population, and rodent community characteristics in Entre Ríos, Argentina between 2004 and 2015 using GLMs [51]. In combination with GIS, hantavirus risk maps were created for this region. The HPS infection risk increased with tree cover and decreased with distance to rivers. Climatic factors assessed included annual mean temperature and annual precipitation. Land cover data were obtained from the MODIS Vegetation Continuous Fields, which provided estimations of surface vegetation cover as gradations of percentage tree cover, nontree vegetation, and bare soil. Three separate response variable models were done. A positive association was found with human hantavirus infection risk and areas with a high percentage of tree cover and locations near rivers. No association of human hantavirus infections with annual precipitation and mean annual temperature was observed.

#### 2.2.3. Chile

A single study (1/13, 7.7%) was conducted in Chile investigating the association of climatic factors and human hantavirus infections (Table 1 and Figure 2). Nsoesie et al. examined the relationship of 667 confirmed HPS cases between 2001–2012 in Chile with temperature, precipitation, and humidity [53]. The models utilized in this study included ARIMA and regression with ARIMA errors to investigate the relationship between HPS cases and temperature, rainfall, and humidity. Only lags of up to four months were considered, and humidity was excluded from modeling. Six different models were developed, including a baseline model that used past HPS case incidence to forecast future HPS cases and the other models included climatic factors as covariates. The quality of the statistical models was assessed using an AIC metric and prediction accuracy by the coefficient of variation (R^2^) and root mean square error (RMSE). HPS epidemic peaks followed peaks in mean temperature whereas a correlation of troughs in HPS cases with periods of increased precipitation and relative humidity was observed. Of the five models selected, the model with lagged rainfall (one and four months) and the models with precipitation were the best fits, indicative of this climatic factor as a critical predictive climatic factor. Limitations to this study included the resolution of climatic factors, the exclusion of the influence of rodent host population distribution and underreporting of HPS in high-risk rural areas. In conclusion, this study provided an association of HPS cases with precipitation and temperature and is robust given the data resolution and availability. Future updates to the modeling approach can enhance sensitivity and prediction accuracy since risk is an evolving entity and constantly changing, thus risk management should likewise mirror such fluidity.

#### 2.2.4. Bolivia

A single study (1/13, 7.7%) was conducted in Bolivia investigating a hantavirus outbreak, which also observed the impact of a climatic factor on human hantavirus infections (Figure 2, Table 1 and Table 2). Montgomery et al. assessed the epidemiological characteristics and the public health risks involving HCPS cases in Santa Cruz, Bolivia during a 2002 hantavirus outbreak [47]. Intense precipitation observed during the outbreak year was higher than in the two preceding years and was associated with the high hantavirus infection rate recorded during the outbreak.

#### 2.2.5. Paraguay

A single study (1/13, 7.7%) was conducted in Paraguay investigating a hantavirus outbreak, which also observed the impact of climatic factors on human hantavirus infections (Figure 2, Table 1 and Table 2). Williams et al. examined HCPS patients in Paraguay during 1995, and an analysis of climate factors found rainfall in May 1995 was tenfold greater than that observed in the preceding 11 years [48]. This study investigated an HCPS outbreak during 1995–1996 in Paraguay which involved 17 human cases. Given the low number of HCPS cases and the shorter duration of study (~1 year) this study was evaluated as weak. Nonetheless, it represents the sole study from Paraguay investigating the association of a climatic factor with HCPS disease. Future modeling studies would be most helpful to elucidate the influence of climate on hantavirus disease in Paraguay.

#### 2.2.6. Panama

A single study (1/13, 7.7%) conducted in Panama investigated a single hantavirus outbreak which also observed the impact of a climatic factor on human hantavirus infections (Table 1 and Table 2 and Figure 2). Bayard et al. reported the association of 2–3-fold higher seasonal rainfall with the 1999–2000 hantavirus outbreak (strain) in Panama [46]. This outbreak involved nine persons with notable morbidity but fortunately no mortalities. The length of the study spanned only 1999–2000, the length of the outbreak, but did not extend any further except for the analysis of rainfall data from the two preceding years. This study is thus weaker than other included studies in providing strong associations with climatic factors. Its inclusion facilitates a wider discussion outside of South America and the Caribbean into Central America regarding climatic factors associated with human hantavirus infections.

#### 2.2.7. Barbados (Caribbean)

Barbados is the only country (1/13, 7.7%) in the Caribbean where human hantavirus infections over a specified time interval have been identified (Table 1 and Table 2 and Figure 2). Douglas et al. investigated 852 human hantavirus cases between 2008–2016, which often had milder symptoms without the full clinical presentation consistent with HPS, HFRS or HCPS [56]. The association of hantavirus cases and the rainy season (June to November) was observed with the peak of hantavirus cases observed in August to September with a potential 3-month lag with the start of the rainy season. Other co-factors associated with increased hantavirus cases included age, gender, and geographical location (urban areas). The statistical analysis conducted involved the use of 95% confidence intervals. No modelling of hantavirus infection risk was attempted in this study, but it would have been valuable for predicting future epidemics based on climate change scenarios. Rodent studies in Barbados have demonstrated serological evidence of hantavirus infection from various parishes of the island [9,57]. Hantavirus infection prevalence among rodents could be incorporated into future models and the creation of hantavirus infection risk maps. However, more intensive rodent studies with larger sample sizes, greater rodent diversity, and a longitudinal design would lend more accuracy prior to inclusion into such modelling studies.

## 3. Discussion

We aimed to examine the associations of climate (including seasonality) factors with the occurrence and risks of human hantavirus infections in Latin America and the Caribbean. Multiple factors including climatic, geospatial, abiotic, and biotic factors were associated with increased human hantavirus infection risks in LAC. 

### 3.1. Rainfall or Precipitation Factor

This was the common climatic factor (75%) consistently positively associated with increased hantavirus infection risk in LAC among the selected studies. This factor was associated with a variable lag period of one to four months, which could be related to the influence of rainfall in creating ideal conditions for sustained rodent host population growth. Studies in other regions also support the association of rainfall/precipitation with hantavirus infection risk including Asia, North America, and Europe [8,55,58,59,60]. A study providing serological evidence of human hantavirus infections in Barbados using data from 2008 to 2016 observed increased human hantavirus transmission during the wet season compared to the dry season [56]. In Latin America, the ENSO phenomenon, and its influence on the temporal dynamics of rainfall is an important consideration. La Niña ENSO events coincide with below average SST leading to wet episodes and increased risk of hantavirus outbreaks as observed during 2010–2011 in Barbados [56], in southern Argentina [50], and other countries in the LAC region [61,62,63,64]. 

Climate effects from rainfall include floods and droughts which can alter rodent host population sizes and their distribution through displacement. Extended wet periods can increase soil moisture, the proliferation of vegetation and food for rodents, which facilitates burrowing and breeding, population increases, and fighting among adult male rodents, which increases hantavirus transmission among rodents from wounding [65,66,67,68,69]. Displaced rodent populations can lead to higher human-rodent contact rates, resulting in a higher risk of human hantavirus infections. Droughts can reduce predation risks for rodents and lower food availability; however, the storage of grains during droughts can also attract rodents. Projections from global circulation models (GCMs) and regional circulation models (RCMs) suggest a general drying trend in the Caribbean with between 20–35% and changes in rainfall extremes, with more heavy rainfall events and more extended drier periods [70]. These projected dry periods followed by intense rainfall events can indicate increased future human hantavirus infection risks for LAC countries [62,63]. 

Some selected studies showed a negative association of rainfall with human hantavirus infections in LAC [44,54]. This could be due to drier conditions and lower food availability causing rodents to forage closer to human habitats in search of food (stored grains and agricultural produce), thus increasing the risk of human-rodent contact [44]. El Nino, an ENSO phenomenon, is associated with reduced rainfall and warmer temperatures. Drier conditions could result in dustier environments, increasing the risk of human inhalation contact with aerosols contaminated by infected rodent urine and or faeces. Additionally, given the low number of selected studies (15.4%) with this negative association of rainfall and HPS cases, other possible factors are likely involved. 

### 3.2. Temperature Factor

Temperature was the most negatively associated climate factor with hantavirus disease among the selected studies but similar rates of positive and negative associations of temperature with HCPS or HPS cases were observed. Temperature can influence rodent abundance and hantavirus disease risk as it promotes vegetation growth, reproduction and survival rates of small rodents [71,72,73], which all affect the survival time of infectious hantaviruses in the environment [74]. Several studies have shown an association of increased temperatures and hantavirus disease [66,75,76], as increased temperatures can also increase aerosol generation, respiratory rates of both humans and rodents [77,78,79]. However, the daily activity of Neotropical rodents decreases with rising temperatures but can increase in combination with rainfall [80,81,82]. High temperatures create unfavorable environments for virus survival but can also cause rodents to seek shelter from heat stress in human habitats increasing hantavirus infection risk [60]. Conversely, low temperatures can prolong virus survival outside the host, which remain infective even without direct inter-rodent contact, or rodent-human contact [74]. 

Thus, the role of temperature both on rodent host activity and virus survival is critical in human hantavirus transmission. Climate projections for the Caribbean indicate that temperatures in the region will be warmer by between 1.0°C and close to 3.5°C [75]. The implication of this warming trend is an increase in evapotranspiration, which reduces soil moisture and, in turn, plant productivity. Excessive warming, heat waves and low precipitation can result in drought conditions, wildfires, and land degradation, which leads to rodent displacement and increased rodent-human contact and the generation of dust aerosols [34,76,83,84,85,86]. 

Wildfires impact climate with CO_2_ emissions and increased particulate matter (PM) levels, which can contribute to the global warming effect potentially exacerbating the effects of climate change in LAC regions. [83]. A few studies have found an association of PM levels with human hantavirus infections in the USA and South Korea [87,88]. Wildfires (brush and sugarcane fires) are thus a potential abiotic factor influencing rodent host distribution and lowering species richness enhancing human-rodent contact risk and human hantavirus infection risk. Future studies should examine the association of temperature and wildfires on the incidence of human hantavirus cases in LAC. 

### 3.3. Humidity Factor

Inhalation of hantavirus infected aerosols which are contaminated via infected rodent excreta is the main route of human infection [74]. Humidity can affect aerosol formation and aerosol dynamics [89,90,91], thus potentially influencing hantavirus transmission and its association with increased risk of hantavirus transmission to humans [74,92,93,94,95]. However, this climatic factor was not extensively explored by all of the selected studies, though the single study that did found a negative association with increased hantavirus risk [53]. Humidity can also impact on plant and vegetation growth, particularly in warm climates like those encountered in the Neotropics [96,97]. This can influence food availability for rodents, increase breeding potential and likely increase rodent population sizes.

### 3.4. Multiple Co-Factors

Geospatial, social, abiotic, and biotic factors such as location, land use management, farming activity, rodent host distribution, habitat type, location away from roads, and human development index (HDI) have been associated with human hantavirus infections among the selected studies in Latin America and the Caribbean. 

The main risk factors in biosecurity can be classified as human, environment, and infrastructure and this can be applied to human hantavirus infections in LAC. Human and socioeconomic factors were identified as being positively associated with hantavirus infections among the selected studies and included HDI, age, gender, social vulnerability, residential location, and population density [17,45,50,51,56]. This highlights the need to include interdisciplinary approaches to future hantavirus infection-climate risk modelling studies and other infectious disease-climate risk modelling studies. The exposure of humans to infected rodents is likely to be complex, thus multivariate models which incorporate several key factors along with climatic factors will likely be more representative of reality and possibly contribute to greater accuracy. 

Two key environmental factors include rodents and land use management. The association of land use management and human hantavirus infections has been observed previously including the proximity of agricultural activity such as planted pasture, sugarcane cultivation and natural forests [98,99]. In addition, the location of animal farms (horse, chicken, pigs, etc.) can encourage the proliferation of wild rodents, possibly attracted to the animal feed stores they can consume, which increases the risk of rodent-human contact and human hantavirus infection risk particular for persons involved in farming activities [12,100,101,102]. In LAC, sugarcane cultivation has been known to influence rodent population and activity due to lower species richness and can lead to increased human contact and increased risks of hantavirus transmission [21,103].

Two biotic factors that strongly influence human hantavirus infection in LAC are rodent host distribution and density. The expansion of agricultural lands has been related to a decrease in meso- and large predators, favouring a greater abundance of generalist rodents infected with hantavirus since these rodent hosts can use monocultures as corridors for nesting and as foraging sites [17,23,55]. This increases the potential risk of human interaction with hantavirus infected rodents [17,104]. Other Neotropical vertebrate species have been shown to be infected with hantaviruses including bats and opossums [23,24,25], while the presence of shrews, another potential vertebrate host, in LAC is noteworthy. They can potentially shed hantavirus in their excreta and be involved in the maintenance, circulation, and transmission of hantaviruses in LAC. Therefore, future studies on hantavirus ecology in LAC should include these other potential vertebrate hosts to elucidate their role in human hantavirus disease risk.

Infrastructure is another factor that can influence hantavirus transmission. There are four factors involved that generate biosecurity risks: trade, travel, tourism and transport [105]. Maritime trade and transportation may also influence hantavirus risk in the LAC region, as it has in spreading the Seoul hantavirus (SEOV) worldwide [106,107,108]. Sewers and stormwater drainage systems can harbour rodents, particularly in urban areas with poor solid waste management, increasing hantavirus infection risks [109,110,111]. Travel is another factor that could influence hantavirus transmission in LAC countries as travel-related human hantavirus infections in LAC have been reported from Cuba [13] and Panama [112]. A review of imported HFRS cases revealed the occurrence of 23 separate travel- or immigration-associated HFRS cases in the published literature [113]. In Barbados, there may also be the possibility of the importation of a hantavirus case from the UK [56]. These biotic factors of land use, rodent host distribution and density, infrastructure, maritime trade, and travel are all influenced by climate and can either increase or decrease human hantavirus infections or infection risks in LAC. Future research should consider them in predictive model development. 

### 3.5. Temporal and Spatial Modelling Approaches 

The inexorability of climate change and its inextricable link to infectious diseases necessitates modelling to provide quantitative risk metrics. These risk metrics are of the utmost importance to decision-makers in public health who are faced with making difficult decisions in a data scarce region that is one of the most vulnerable to the effects of climate change. While there have been modelling studies in Latin America aimed at elucidating the spatiotemporal dynamics of HPS/HCPS incidence rates and risks, there are no known studies for the Caribbean. Table 1 and Table 2 provide a summary of the modelling studies found in the peer-reviewed literature for the LAC region. The main modelling approaches used in these studies were the ARIMA and GLM approaches, and Bayesian and ecological niche models. The most common climatic explanatory factors used in the models are temperature and rainfall, while the major non-climatic co-factors considered were land-use related. 

Commonly used performance metrics to assess the quality of models were the coefficient of correlation (R^2^), the Akaike information criterion (AIC), area under the curve (AUC), and root mean square error (RMSE). The purpose of a study largely drives the modelling approach utilised. For investigating temporal infectious disease dynamics, ARIMA models are appropriate [53], whereas GLM and ecological niche models are targeted for a spatial understanding of risk [44,50,51]. An ARIMA model was used to estimate and forecast HPS activity in Chile [53]. The predictor variable was the number of HPS cases, while the explanatory variables included temperature, rainfall, and humidity. The ARIMA approach demonstrated promising results, showing a correlation between outbreak peaks preceded by mean temperature peaks capturing the temporal behaviour of rainfall and relative humidity correlated with confirmed HPS cases [53]. Given the relative simplicity of the ARIMA approach and current data availability, this class of model constitutes perhaps the most feasible starting point for the Caribbean. The risk maps generated from modelling could be critical in assisting public health authorities to better target surveillance efforts. The main challenge that stymies the ability to take the lessons learned from spatial risk modelling in Latin America and apply them to the Caribbean is data availability. To the best of our knowledge, the data available for hantavirus infections in the Caribbean support studies on temporal dynamics is very limited; furthermore, there is a lack of information required to do robust spatial modelling studies. 

### 3.6. Selected Studies: Limitations

There are limitations noted in the selected studies including the resolution of climatic factors (typically monthly or weekly), low HPS cases numbers, short duration of study, source of data (climate databases rather than local climatic datasets), partial absence of rodent host population and hantavirus infection dynamics data, absence of other specific climatic/abiotic factors such as sea surface temperature (SST) and particulate matter levels. These could all impact on the accuracy of predictive models for human hantavirus infections. The bibliographic search is also limited by the existing peer reviewed studies present on the databases searched namely PubMed and Web of Science. Other pertinent studies not currently indexed at the time of conducting the bibliographic search may exist so the list of selected studies may not be all inclusive.

### 3.7. Knowledge Gaps & Future Research

There is not enough impact-based forecasting for infectious diseases and climate conducted in the Caribbean, and given the current predictions for climate change, mitigation plans for potential impacts will be critical in reducing the damage and negative impacts felt in multiple sectors. This would also extend to Latin America, though more research has been conducted here than in the Caribbean. More research is needed to understand the influence of climatic factors and future climate change scenarios on infectious disease transmission dynamics and disease risks. The role of trade and traffic in increased hantavirus infection risks, probably through increased food availability [114,115], and even introducing non-native rodent hosts into virgin areas, has not been fully explored in LAC. Additionally, the role that the built environment and infrastructure (leaking water distribution networks, vegetative growth, and increased food availability to rodent hosts), international trade and travel can play in increased human hantavirus infection risk requires more work to better simulate real world scenarios with the various cultural and environmental nuances in LAC. The impacts of other climatic factors such as particulate matter (PM_2.5_ and PM_10_) levels on hantavirus infection risks, though explored with studies in other regions such as Asia and North America, have not been explored in LAC [87,88]. This requires investigation in this region where significant Saharan dust activity, indiscriminate burning of refuse, vehicular exhaust and brake dust, all significant generators of particulate matter, are commonplace [116,117,118,119,120].

## 4. Materials and Methods

### 4.1. Bibliographic Search

**Figure 3:**
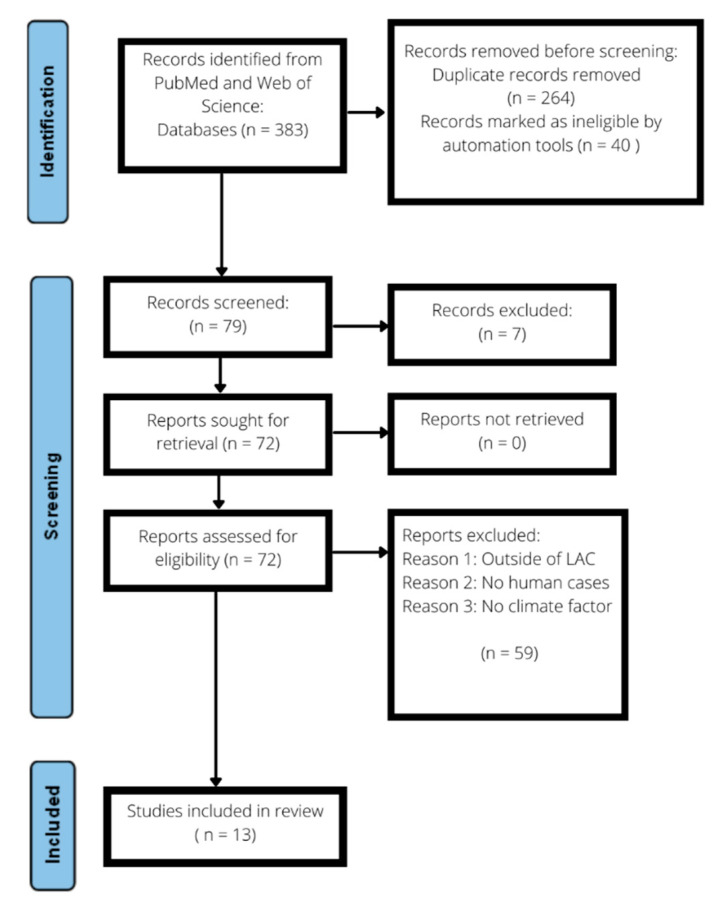
PRISMA bibliographic search (MEDLINE and Web of Science), screening, and study selection flowchart.

Using a previous method based on patient, intervention/exposure, comparator, outcomes study design (PECOS) criteria and a PRISMA checklist, we conducted a systematic review by performing a bibliographic search for all relevant peer-reviewed journal publications on climate and hantavirus infections in Latin America and the Caribbean using a pre-established search strategy (Table 3 and Figure 3) [8,46]. For this systematic review we defined the population of interest as humans of all ages and sexes, the exposure as short- and long-term climate variability, and the outcome as HFRS, HPS, HCPS or serologically or reverse transcriptase polymerase chain reaction (RT-PCR) confirmed human hantavirus infections.

### 4.2. Selection Criteria

For the defined inclusion criteria, only peer-reviewed studies involving human hantavirus infections, available as full-text articles, with relevant abstracts or study titles (with climate and human hantavirus infections) were considered (with no limits on age or gender). Exposures were variability of different climatic factors over the short- and long- term (a minimum of a one-year period of observation), and finally the outcome was acute hantavirus infections with limitations to Latin America and the Caribbean. This one year minimum was established to mitigate against the impact of seasonality and short-term weather changes. However, instances of hantavirus outbreaks with an association with rainfall or rainfall seasonality were also included due to the sporadic nature of hantavirus outbreaks in LAC, the few studies conducted, and to highlight the need for more future studies in wider parts of the LAC region. An acute hantavirus infection was defined in accordance with the CDC definition of a non-HPS case and an HPS case [121]. 

The exclusion criteria for the systematic review included (1) non-primary research studies, (2) studies outside of the LAC region, and (3) studies investigating only hantavirus infections in rodent hosts and associations with climate factors but not human hantavirus infections or human hantavirus infection risk. 

Studies investigating the association of climatic factors and acute human hantavirus infections and or disease in Latin America and the Caribbean were all included for collection and analysis.

### 4.3. Search String

The development of a suitable search string was completed inclusive of the search terms framed by the relevant inclusion and exclusion criteria [122]. This search string was designed as follows: (climate OR hantavirus OR human) (climate OR weather OR drought OR season* OR rain* OR precipitation OR flood OR temperature or humidity AND hantavirus OR hemorrhagic fever with renal syndrome OR HFRS OR hantavirus cardiopulmonary syndrome OR HCPS OR Hantavirus pulmonary syndrome OR HPS) similar to a previous study [8]. Searches were conducted on MEDLINE using PubMed and Web of Science databases. Notably, this bibliographic search lists studies in these databases updated as of 15th July 2021. 

### 4.4. Study Selection and Quality Assessment

Two independent reviewers selected the studies for which full texts were obtained and the two readers then decided independently about the final inclusion of articles. In cases of disagreement, an arbiter was involved. In addition, using relevant review journal articles on climate and human hantavirus infections and additional studies meeting the inclusion criteria were added to the list of selected studies. Each methodology of the selected studies was analysed for quality, including risk of bias using Scottish Intercollegiate Guidelines Network (SIGN) and Critical Appraisal Skills Programme (CASP) guidelines. The outcome measurement was included in the checklist, with higher ratings for serologically confirmed hantavirus cases in preference to self-reported or symptomatic diagnoses. The influence of confounders was also accounted for as previously done [8]. Assessments were conducted and recorded for each selected study using a three-point scale prescribed by SIGN which indicates ++ as very high quality, + as high quality and − as low quality. This quality rating followed the range of fulfillment of the criteria regarding validity of results. The complete checklist is shown in Appendix A and the scoring system is available in Table S1.

Data from these selected studies were extracted independently to compile an evidence table (Table 1) which provided pertinent information of study location, number of human cases, study period, exposure, outcome, and notable results related to climate and related co-factors. In addition, metrics and statistical assessments used to assess the models and analyses and the assigned quality of each study were noted (Table 2). Given the varied nature of methodologies utilized in the selected studies and varied geographical locations, a qualitative synthesis of study data was conducted rather than a quantitative analysis that is done for meta-analysis. Other associated co-factors included other social, demographic, socioeconomic, geospatial, and biotic factors. 

For human hantavirus infections, a serological and or molecular test confirmation of infection was the outcome, and the effect measure was variable given the heterogeneity of the study methods used in the selected studies. For missing summary statistics, the evaluation of the study design, outcome and results were used to deduce the vigour of the study under examination to determine aptness and accuracy.

## 5. Conclusions

Climatic factors, namely rainfall or precipitation, humidity, and temperature, have been linked to human hantavirus cases in Latin America and one Caribbean country. Rainfall is the climate factor most often positively associated with human hantavirus infections in LAC. However, more research with robust examination of longer temporal spread and enhanced hantavirus monitoring and surveillance in humans and rodents would aid in refining the predictive power of models. The climate change scenarios advanced by the Intergovernmental Panel on Climate Change (IPCC) indicate more climatic variability and intensity for the LAC region. A more thorough understanding of both the role of host population dynamics, other abiotic and biotic factors, and hantavirus infections due to the influence of climate is imperative to achieve better modeling of human hantavirus infections and the risk prediction of future outbreaks and or epidemics. This is very useful in LAC, where such hantavirus outbreaks or epidemics can occur simultaneously with other infectious disease epidemics like dengue, Zika and Chikungunya fevers.

This systematic review was registered with PROSPERO under the registration number CRD42021283357 on 17 December 2021.

## Figures and Tables

**Table 1:** Analysis of selected published peer-reviewed studies.

Study	Quality	Study Location	Study Design	Time Period	Human Cases (n)	Climatic Factors	Outcome	Co-Factors	Statistical Methods	Results
Donalisio (2008)	+	Brazil	Ecological	1993–2005	80	Rainfall (mm)	HPS cases	Spatial analysis	None	Higher HPS incidence observed in drier months and increased access to food sources by rodents (Harvesting and grain storage and sugar cane cultivation).
Donalisio (2011)	++		ENM	1993–2008	288	Winter precipitation	HPS cases	GIS, topography, men > 14 years old and EVI	None	Winter precipitation increased in the dry season and EVI was associated with HPS disease incidence.
Prist (2016)	++		Bayesian model	1993–2012	207	Rainfall and temperature	HCPS risk	HDI, men > 14 years old, sugarcane, population at risk and landscape	t-test, one-way ANOVA, and Tukey’s MCT	HDI and sugarcane cultivation were associated with HCPS cases in the Cerrado whilst males > 14 years old, HDI, sugarcane cultivation and temperature were associated with HPS cases in the Atlantic Forest.
Prist (2017)	+		Bayesian model	2000–2010	?	Temperature, precipitation, RCP4.5 and RCP8.5	HCPS risk & HCPS cases	Sugarcane cultivation	Moran’s I, Queen’s case neighbourhood relation	A positive association was observed with increased temperatures & sugarcane cultivation and HCPS risk and HCPS cases.
Muyalert (2019)	++		Zero inflated model and temporal term (rw2) + Besag Intrinsic Conditional Auto-Regressive (ICAR) spatial model	1993–2016	1758	Temperature and precipitation	HCPS risk & HCPS cases	Host diversity, social vulnerability, and land use change (sugarcane)	INLA	A positive effect was observed with size of population at risk, social vulnerability, rainfall, host diversity, rural workers, land use (sugarcane, maize, and forest cover) on HCPS risk and HCPS cases whilst a negative effect was observed with temperature.
Andreo (2014)	++	Argentina	GLM and MaxEnt models	1995–2009	149	Climate and precipitation related factors	HPS cases	Altitude, rodent host, vegetation, & GIS	Kruskal-Wallis and pairwise Pearson correlation	A positive association of precipitation, forested and scrubland habitats with HPS cases was observed.
Vadell (2019)	++		GLM	2004–2015	60	Rainfall and temperature	HPS cases	Area distance from roads, vegetation, topography, number of human inhabitants and reservoir host population	Kappa index, VIF analysis, bootstrap procedure, and residual plots	A positive association with of HPS cases with areas with high percentage of tree cover and locations near rivers was observed. No association with annual precipitation and mean annual temperature was observed and human hantavirus cases.
Ferro (2020)	++	Argentina	ARIMA & dynamic regression models	1997–2017	902	Rainfall and temperature	HPS cases	None	Augmented Dickey-Fuller Test, AICc, RMSE & Ljund-Box test	A positive association of HPS cases with rainfall and temperature was observed with notable delay or lags ranging from two to six months.
Montgomery (2012)	-	Bolivia	Epidemiology	2002	45	Rainfall	HPS cases	Age and farming	None mentioned	A possible association of HPS cases with monthly precipitation levels exists.
Nsoesie (2014)	++	Chile	ARIMA and regression with ARIMA errors	2001–2012	667	Precipitation, temperature & humidity	HPS cases	None	R^2^ and RMSE	Positive associations of HPS cases with peaks in temperatures and HPS troughs with precipitation and humidity levels were observed.
Bayard (2004)	−	Panama	Outbreak investigation	1999–2000	9	Precipitation	HPS cases	None	None mentioned	A positive association of HPS cases with increased rainfall (two to threefold) during September/October 1999 (outbreak year) was observed.
Williams (1997)	−	Paraguay	Outbreak investigation	1995–1996	17	Precipitation	HPS cases	None	None mentioned	A positive association of HPS cases with increased rainfall (10-fold) during the 1995 (outbreak year) was observed.
Douglas (2021)	+	Barbados	Cross-sectional epidemiology	2008–2016	862	Rainfall (seasonality)	Hantavirus cases *	Gender, age and urban location	95% CI	A positive association of human hantavirus infections with rainy season, age, gender, and geospatial location was observed.

Key ?—not specified; ++ very high quality study; + high quality study; − low quality study; *—hantavirus cases refer to laboratory confirmed hantavirus infect ions via ELISA testing not via syndromic surveillance (the strain(s) involved is/are currently unknown); Akaike information criteria (AICc); Analysis of variance (ANOVA); Autoregressive Integrated Moving Average (ARIMA); confidence intervals (CI); Ecological Niche model (ENM); EVI (Enhanced Vegetation Index); geographical information systems (GIS); Generalised linear model (GLM); Human development index (HDI), Hantavirus pulmonary syndrome (HPS); Hantavirus cardiopulmonary syndrome (HCPS); Integrated nested Laplace approximations (INLA); Tukey’s multiple comparison tests (MCT); Representative Concentration Pathway 4.5 (RCP4.5), Representative Concentration Pathway 8.5 (RCP 8.5); Root mean square error (RMSE); Variance inflation factor (VIF); Coefficient of variation R^2^.

**Table 2:** Analysis of selected published peer-reviewed studies and the relevant metrics used to assess model performance and their respective numerical values for the best and the worst candidate models.

Study	Quality	Study Location	Study Design	Statistical Methods	Metric	Best Model Value	Worst Model Value
Donalisio (2008)	+	Brazil	Ecological	None	N/A	N/A	N/A
Donalisio (2011)	++		ENM	*t*-test	*p*-value	0.02	0.0001
Prist (2016)	++		Bayesian model	*t*-test, one-way ANOVA, and Tukey’s MCT	*p*-value	0.9231	0.0626
Prist (2017)	+		Bayesian model	Moran’s I, Queen’s case neighbourhood relation	SD of risk values	3.4	3.8
Muyalert (2019)	++		Zero inflated model and temporal term (rw2) + Besag ICAR spatial model	INLA, Moran’s I, cross validation procedures	CPO	0.58 *	0.36
Andreo (2014)	++	Argentina	GLM and MaxEnt models	Kruskal-Wallis and pairwise Pearson correlation	AIC	76.07	100.42
Vadell (2019)	++		GLM	Kappa index, VIF analysis, bootstrap procedure, and residual plotsAugmented Dickey-Fuller Test, AICc, RMSE & Ljund-Box test	Kappa index	0.58	0.44
					Explained deviance	70.5%	61.2%
Ferro (2020)	++		ARIMA & dynamic regression models		AICc	97.74	291.4
					RMSE	0.91	0.78
					R^2^_adj_	0.71	0.54
Montgomery (2012)	-	Bolivia	Epidemiology	None mentioned	N/A	N/A	N/A
Nsoesie (2014)	++	Chile	ARIMA and regression with ARIMA errors	Coefficient of variation R^2^ and RMSE	AICc	437.62	444.98
					RMSE	2.421	2.774
					R^2^	0.594	0.551
Bayard (2004)	−	Panama	Outbreak investigation	None mentioned	N/A	N/A	N/A
Williams (1997)	−	Paraguay	Outbreak investigation	None mentioned	N/A	N/A	N/A
Douglas (2021)	+	Barbados	Cross-sectional epidemiology	95% confidence intervals (CI)	N/A	N/A	N/A

Key ++ very high quality study; + high quality study; − low quality study; N/A—not applicable; R^2^—r squared-coefficient of determination; R^2^_adj_—r squared adjusted is r squared that has been adjusted to the number of predictors in the model; *—This is an optimised mean to improve CPO values from a zero-truncated Poisson model; Akaike information criteria (AICc); Analysis of variance (ANOVA); Autoregressive Integrated Moving Average (ARIMA); Cross validation procedures (CPO); Ecological Niche model (ENM); Generalised linear model (GLM); Intrinsic Conditional Auto-Regressive (ICAR); Integrated nested Laplace approximations (INLA); Maximum entropy (MaxEnt); Tukey’s multiple comparison tests (MCT); Root mean square error (RMSE); Standard deviation (SD); Variance inflation factor (VIF).

**Table 3:** PECOS criteria followed in the review.

Parameters	Inclusion Criteria	Exclusion Criteria
Population	People with acute hantavirus infection or HPS/HCPS/HFRS	Only animal hantavirus infections
Exposure	At least one climate factor	No climate factors
Comparators	N/A	
Outcomes	Hantavirus case/infection/risk/incidence	Rodent density/distribution
Study design	Observational, epidemiological, retrospective, predictive modelling study design	Prospective study design

Key N/A—not applicable; PECOS—patient, intervention/exposure, comparator, outcomes, study design; hantavirus pulmonary syndrome (HPS); hantavirus cardiopulmonary syndrome (HCPS); haemorrhagic fever with renal syndrome (HFRS).

## Data Availability

Not applicable.

## Supplementary Materials

The following are available online at https://www.mdpi.com/article/10.3390/pathogens11010015/s1. Table S1. Scoring system for quality assessment of selected studies.

## Appendix A

The appendix is a summary of the checklist of the relevant aspects of each study under review for the assessment of several quality criteria to permit the scoring of a grade. Please see also Table S1.

Box A1 Checklist for Quality Assessment of Selected Studies in the Systematic Review.

A. **Validity of Study Results**
  1. Was a clear focus issue addressed?—Yes/Unsure/No
    Follow on and ask some of these other questions to evaluate the rating.
  2. Which study design was utilized?
  3. Was the appropriate method used to answer the question posed?
  4. Was/Were the climate exposure factor(s) accurately measured to minimize bias?
  5. Was the outcome accurately measured to minimize bias?
  6. Were all the important confounding factors identified?
  7. Are these confounding factors addressed in the design and or analysis?
B. **What are the Study Results?**
  1. What are the results of this study?—Yes/Unsure/No
    Follow and ask some of these other questions to evaluate the rating.
  2. What is the level of precision of the results? What is the level of precision for the risk estimate used?
  3. Are the results believable based on statistics?
  4. How was this study funded?
C. **Applicability of Study Results to This Systematic Review**
  1. Does this study answer the question posed by this systematic review?—Yes/Unsure/No
    Follow on and ask some of these other questions to evaluate the rating.
  2. What is the level of precision of the results? What is the level of precision for the risk estimate used?
  3. Are the results believable?
