# Supplementary material for: Influence of Climatic Factors on Human Hantavirus Infections in Latin America and the Caribbean: A Systematic Review
Source: Pathogens. 2021 Dec 23;11(1):15. doi: 10.3390/pathogens11010015 (PMC8778283; doi:10.3390/pathogens11010015)
Supplement: Supplementary file 1 [file pathogens-11-00015-s001.zip › pathogens-1403204-supplementary.pdf]

Table S1. Scoring system for quality assessment of selected studies.

Criteria	-	+	++
**Selection**	No description	Partial description	Full description
**Study design**	No description	Partial description	Full description
**Risk of bias**	No description of bias reduction	Partial description of bias reduction	Full description of bias reduction
**Data collection**	No description/reliable source	Partial description	Full description & reliable source
**Observational time period**	Less than a single year	Single year	Greater than 1 year
**Analysis**	Non-appropriate method for study design (correlation)	Not a fully appropriate method for study design (linear regression)	Appropriate method for study design (Poisson regression)
**Interpretation of factors**	No statistical test or measure	Statistically significant but with no reported measure	Statistically significant with measures
**Funding of study**	No description	Partial description	Full description
**Applicability to review study question**	Not applicable	Partially applicable	Applicable
**Hantavirus diagnosis**	No description	Partial description/suspected cases	Full description/laboratory confirmed cases

Grading: ++ for very high quality studies scoring more than 7 (++); + for high quality studies scoring at least 7 (++); for low quality studies scoring less than 7 (++).
